# Home and Community Based Service Use Among Veterans With Dementia Living in Rural Virginia

**DOI:** 10.1093/geroni/igab046.412

**Published:** 2021-12-17

**Authors:** Jyoti Savla, Mamta Sapra, Lauren Hagemann, Katherine Luci

**Affiliations:** 1 Virginia Tech, Blacksburg, Virginia, United States; 2 Salem Veteran Affairs Medical Center, Salem VAMC, Virginia, United States; 3 Salem VAMC, salem, Virginia, United States; 4 Salem VA Medical Center, Salem, Virginia, United States

## Abstract

Despite the overall expansion of rural Veteran health care facilities, older Veterans in these areas are still underserved and face challenges and barriers to access services. Using data from 60 family caregivers of persons with dementia (PwD; Mean Age = 67 years, Range = 39-84; 92% White; 71% Spouse) we examined the types of home-based and community services they utilized. We also examined reasons that family caregivers provided for not using these services. Next, we applied Andersen's Behavioral Model of Health Services to examine how predisposing factors such as demographics, available resources, and PwD’s needs were associated with the use of services. We found that Veterans living in rural counties had lower access to caregiver support groups, homemaker services, adult day centers, and home-based respite services. The top three reasons for not using services were that the family caregiver chose to do it themselves, the PwD did not want the service or the service provider to help, or it was too far from the caregiver’s home. Regression analysis further showed that caregivers caring for PwDs with greater ADL challenges and memory and behavior problems were more likely to need and utilize paid services. Disparities based on gender, age, and race were also explored. Findings suggest the need to develop effective service promotion strategies and destigmatizing the use of paid services among Veteran families to reduce health disparities in rural regions.

